# Protection against Th17 Cells Differentiation by an Interleukin-23 Receptor Cytokine-Binding Homology Region

**DOI:** 10.1371/journal.pone.0045625

**Published:** 2012-09-19

**Authors:** Wei Guo, Cheng Luo, Chen Wang, Yue Zhu, Xin Wang, Xiangdong Gao, Wenbing Yao

**Affiliations:** State Key Laboratory of Natural Medicines, School of Life Science and Technology, China Pharmaceutical University, Nanjing, People’s Republic of China; Charité, Campus Benjamin Franklin, Germany

## Abstract

Th17 cells have been reported to produce proinflammatory cytokines like Interleukin-17, IL-22, and regarded as important players in various inflammatory diseases. One of the IL-12 cytokine family cytokines, IL-23, composed of p19 and p40 subunit, is known for its potential to promote Th17 development and IL-17 producing, and the IL-23/IL-17 pathway is considered to be potential therapeutic target for autoimmune inflammation responses. Knockout mice deficient in either IL-23 or IL-17 related genes can suppress the allergic responses. Several IL-23 or IL-17 neutralizing agents are being evaluated *in vitro or in vivo* to disrupt the IL-23/IL-17 axis. Herein, we report that prokaryotically expressed soluble IL-23 receptor cytokine-binding homology region as an endogenous extracellular receptor analogue could be a natural antagonist against IL-23/IL-17 axis. We provide evidence that IL23R-CHR can bind to IL-23 in a dose-dependent manner *in vitro*, and block IL-23 signal by IL23R-CHR reducing the RORγt expression, which in turn lowers the expression of IL-17/IL-22, thus protecting naive CD4+ T cells against Th17 development. Together, this study indicates the importance of IL-23 pathway in Th17 development and the negative regulation of Th17 development by IL23R-CHR, and highlights the important roles of the soluble receptor extracellular region in the therapeutic strategy of neutralizing IL-23.

## Introduction

Inflammation is an extremely complex biological response caused by various factors, and the acute inflammatory response is the initial response of the host toward a diverse array of biological stimuli including containment and elimination of microbial invaders [1]. Uncontrolled inflammation has been considered as a pathophysiologic basis for many widely existing diseases in the general population that were not initially known to be linked to the inflammatory response, including cardiovascular disease, asthma, arthritis, and cancer [2], [3].

CD4+ T cells display a critical role in adaptive immunity [4]. When specific antigens are presented on APCs, naïve CD4+ T cells will be subdivided into different subsets of effector cells. On the basis of the cytokines generated by macrophages and dendritic cells (DCs), CD4+ T cells can become different T helper subsets such as Th1, Th2 and Th17 or regulatory T cells [5], wherein Th17 cells are known to be cellular mediators of inflammation in autoimmune diseases [6], playing an important role in the development of multiple sclerosis (MS) and experimental allergic encephalomyelitis(EAE) [7]. Experimental and clinical data have suggested that CNS inflammation could be due to over-reactivation of Th17 cells [8]. Interleukin-17, a proinflammatory cytokine predominantly produced by Th17 cells, promotes inflammation by directly causing tissue injury and enhancing secretion of pro-inflammatory cytokines and chemokines by resident cells [9]. IL-17 serum level is increased in patients with a variety of allergic and autoimmune diseases such as MS and rheumatoid arthritis(RA) [10], suggesting the contribution of IL-17 to the induction and/or development of these diseases. This is supported by the study of the involvement of IL-17 in these responses in animal models of EAE for MS [10], [11], wherein the reactivity was suppressed in IL-17-deficient mice. Up to date, accumulating data support the central role of Th17 cells and IL-17 in inflammatory process and in animal models of autoimmunity or inflammation [12].

IL-23, a new member of the IL-12 cytokine family, playing an important role in stimulating survival and proliferation of Th17 cells and maintaining Th17 effector function [13], [14], is recently identified as a heterodimeric proinflammatory cytokine mainly produced by DCs and activated macrophages [15]. IL-23 shares a common p40 subunit with IL-12, whereas p19 is unique for IL-23 [15]. IL-23 receptor complex is also composed of two chains: the IL-12 receptor β1 chain and the IL-23 receptor chain [16]. IL-23 receptor complex transfers the IL-23 signals to T helper cells and mediates its proinflammatory effects through the activation of Th17 cells that secrete IL-17 [10], [17]. Furthermore IL-23 conducts the development of Th17 cells and promotes chronic inflammation dominated by IL-17, promotes autoimmune inflammation mediated by Th17 cells and has been linked to many human immune disorders [18]. Many pathologic defects found in animal models of autoimmunity are initially associated to IL-12 and Th1 cells but are in fact caused by IL-23 [19]. Knockout mice deficient in either p19 or p19 receptor (IL-23R) develop less severe symptoms of EAE, highlighting the importance of IL-23/IL-23R in the inflammatory pathway. Recently several reports have established the critical function of the IL-23/IL-17 pathway in autoimmune diseases [20]. In the absence of IL-23R, Th17 development is stalled at the early activation stage, leading to less Th17 proliferation and fewer effector Th17 cells [18]. Targeting IL-23, anti-IL-23 therapy can effectively inhibit multiple inflammatory pathways that are critical for driving autoimmune inflammation [21], and IL-23 blockade with neutralizing antibodies or genetic inactivation of the IL-23p19 gene could dramatically protect animals against allergic response [21].

Accumulating evidence shows that chronic inflammation is associated with various diseases. Therefore, control of inflammatory functions of immune cells emerges as a novel strategy to treat or cure many chronic diseases. While TGF-β and IL-6 induce Th17 cells differentiation, IL-23 is expected to promote Th17 cells proliferation and maturation. Therefore in the present study, we obtained a protein containing IL-23R cytokine-binding homology region (CHR) by prokaryotic expression system, to create an IL-23p19 antagonist that specifically blocks the IL-23 signals, interrupts the IL-23/IL-17 axis and ultimately suppresses Th17 development. This strategy was based on a “WSXWS” motif in the extracellular region of IL-23R [16]. And in the study, we investigated the binding ability of IL23R-CHR with IL-23 using Native-PAGE and direct binding ELISA, also evaluated the role for IL23R-CHR in the inducible expression of IL-17, IL-22 and RORγt in activated Th17 cells. After the measurements of the number changes of Th17 cells with IL23R-CHR treatment, the expression level of IL-17 and IL-22, and the mRNA level of IL-17, IL-22 and RORγt, our results demonstrated that IL23R-CHR could be useful against IL-23 signals and rescue the development of Th17. Consequently, IL23R-CHR could be considered as one of the potential agents that neutralize IL-23 and treat IL-23/IL-17 related diseases.

## Results

### Amplification of the IL23R-CHR Gene by PCR

**Figure 1:**
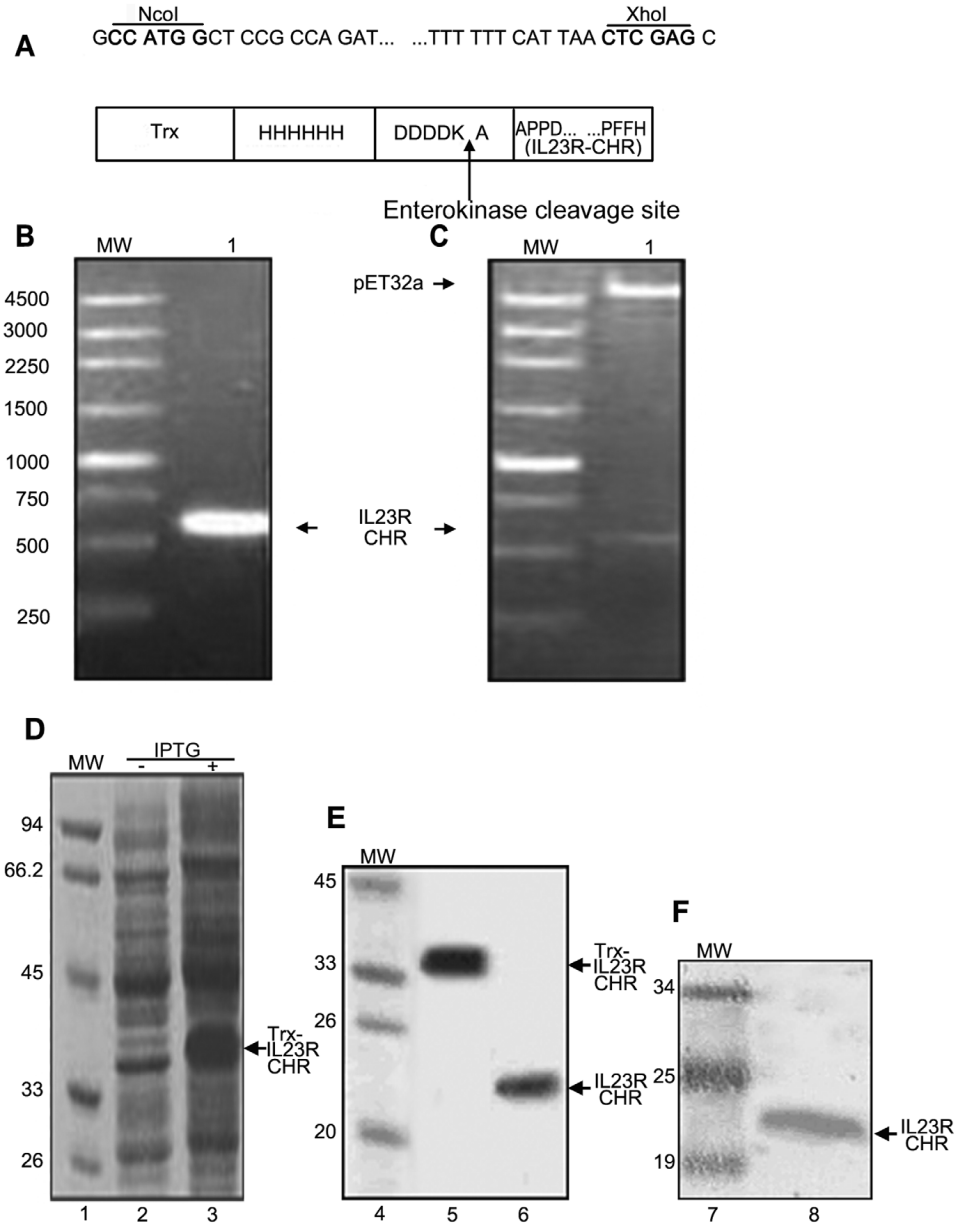
Generation of IL23R-CHR protein. A, Restriction sites are indicated in bold, the translated amino acid and Trx-6×His-DDDDKA tag are shown in the box; B, IL23R-CHR gene from human spleen cDNA library amplified by PCR; C, One pET32a/IL23R-CHR clone digested with NcoI and XhoI and analyzed by agrose gel electrophoresis (1%). MW = molecular weight markers, bp. D, Trx-IL23R-CHR induced by IPTG in *E.Coli* BL21 (DE3), SDS-PAGE (15%), protein bands were stained with coomassie brilliant blue R250 reagent. Lane 2, uninduced bacterial lysate; Lane 3, IPTG induced bacterial lysate. E, Trx-IL23R-CHR purified from BL21 (DE3) lysate and cleaved by enterokinase, Lane 5, purified Trx-IL23R-CHR; Lane 6, purified IL23R-CHR. F, Western blot using mouse mAbs against human IL23R (Lane 8). Lane 1, 4 and 7 in D, E, F: molecular weight markers, KD.

The IL-23R-CHR gene (570 bp) was amplified from human spleen cDNA library, and the amplicon was cloned in frame within NcoI and XhoI restriction sites of the pET32a expression vector, creating a fusion product behind the Trx-hexahistidine-DDDDKA tag sequence, as shown in Figure 1A. The IL23R-CHR can be released from the fusion protein by using enterokinase to cleave the enterokinase cleavage site between “DDDDK” and “A-IL23R-CHR”. Competent *E.coli* BL21 (DE3) was successfully transformed with the recombinant plasmids. The details of the IL23R-CHR gene and recombinant plasmid are presented in Figure 1B and C.

### Expression and Purification of IL23R-CHR

Expression and purification of IL23R-CHR were demonstrated by SDS-PAGE and Western Blot (Figure 1). The Trx fusion IL23R-CHR protein was successfully induced by IPTG in *E.Coli* BL21(DE3) (Figure 1D). The clarified Trx-IL23R-CHR lysate was purified on Ni-NTA column, and IL23R-CHR was released by enterokinase cleavage. The eluted protein was analyzed by SDS-PAGE (Figure 1E). IL23R-CHR (lane 8) was recognized by anti-IL23R mAbs (R&D) by Western blot (Figure 1F), indicating that the purified product was IL23R-CHR (22KD). IL23R-CHR was cloned into pET32a, pET32a containing a N-terminal Trx Tag. The Trx tag was reported to catalyze the formation of disulfide bonds in the cytoplasm and induce the fusion protein more soluble [22], [23], which ensures the expression of soluble, active, properly folded IL23R-CHR, and we used the soluble protein to conduct following investigation.

### Binding of IL23R-CHR to Human IL-23

**Figure 2:**
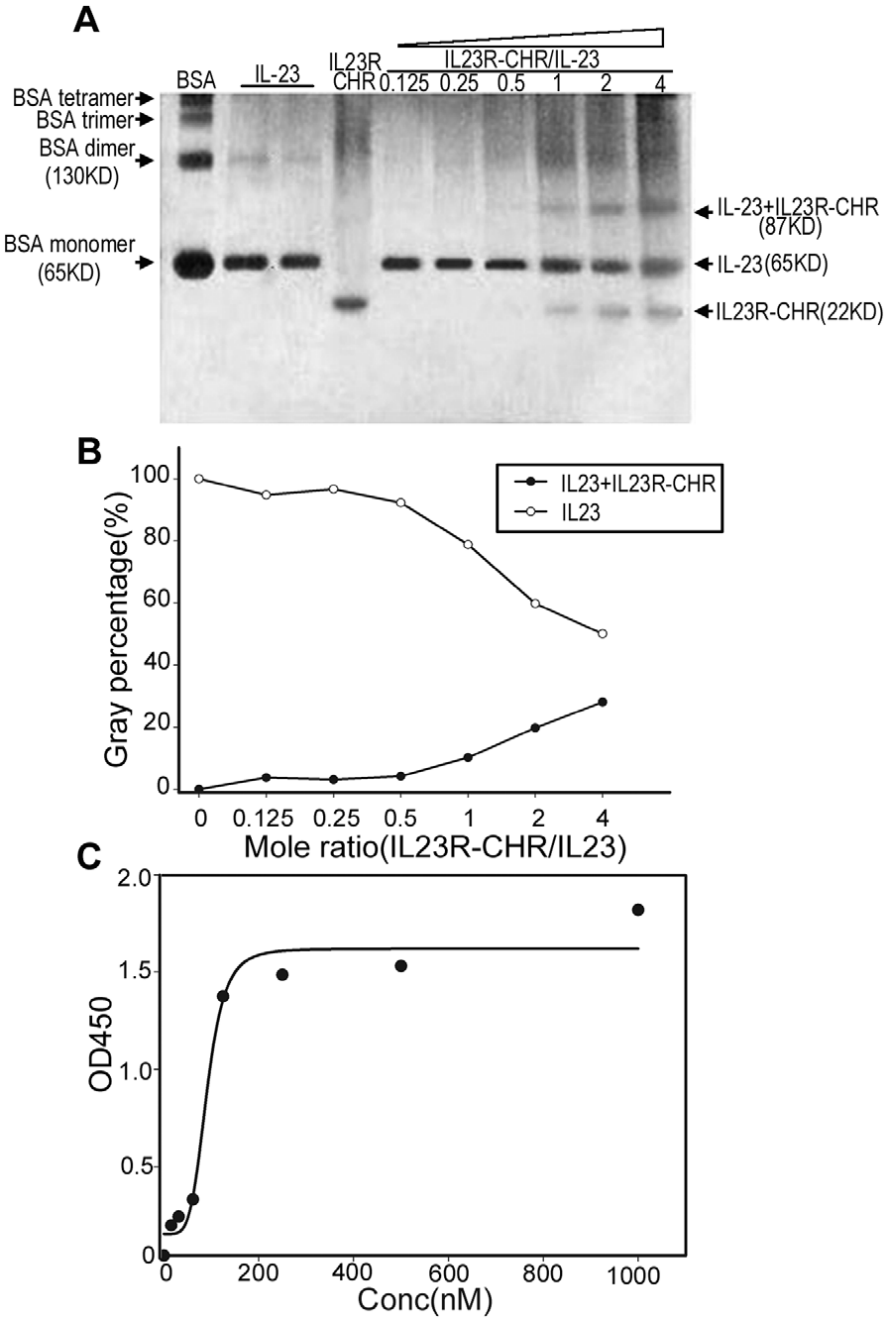
IL23R-CHR binds to human IL-23. A, IL23R-CHR assay by native PAGE, Lane 1,BSA as control; Lane 2 and 3, IL-23 alone; Lane 4, IL23R-CHR alone; Lane 5–10, IL-23 incubated with varied concentrations of IL23R-CHR. B, Results of image analysis of the gel in A. C, IL23R-CHR binds IL-23 in direct binding ELISA.

To investigate the binding of IL23R-CHR to human IL-23, direct binding analysis was performed by incubating 20 ng human IL-23 with IL23R-CHR with a mole ratio varying from 0.125 to 4 (IL23R-CHR/IL23). The IL-23/IL23R-CHR complex was subjected to native PAGE which was then silver stained and analyzed by Gel-Pro analyzer (Media Cybernetics int, USA). The IL23R-CHR induced the increase of IL-23/IL23R-CHR complex in a dose-dependent manner and decreased the amount of IL-23 (Figure 2). To quantify the interaction between IL23-CHR and IL-23, the percentages of IL-23/IL23R-CHR complex, unbound fraction of IL-23 and unbound fraction of IL23R-CHR were determined by Gray scanning respectively.

The direct binding ELISA assay was designed to determine the binding affinity between human IL-23 and IL23R-CHR, and the IC_50_ value was 90 nM (Figure 2C). All the binding assays confirmed that IL23R-CHR can bind to human IL-23, which ensures the biological activity of IL23R-CHR.

### IL23R-CHR Inhibited the Secretion of IL-17a in vitro

**Figure 3:**
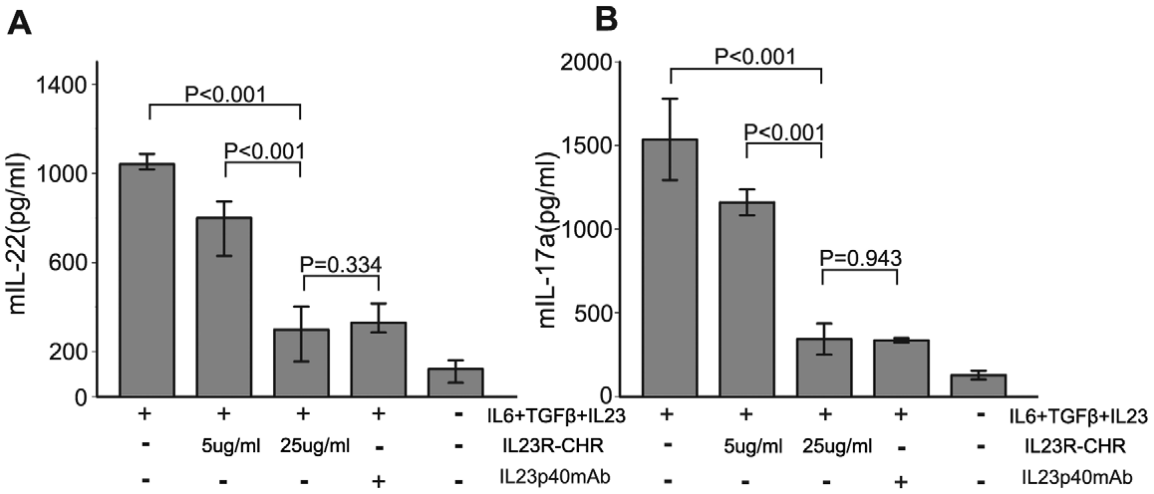
IL23R-CHR inhibits the secretion of Th17 related cytokines. A, IL23p40 mAb and IL23R-CHR suppressed the level of secreted IL-22. B, IL23p40 mAb and IL23R-CHR inhibits the secretion of IL-17a in a dose dependent manner. Naive CD4+T cells were cultivated under the same conditions as for Th17 in the presence of purified IL23R-CHR (5 ug/ml, 25 ug/ml) or IL23p40 mAb (1 ug/ml) for 72 hours. Expression of IL-17a and IL-22 by CD4+T cells was determined by ELISA. Error bars indicate ± SD among duplicate samples from one experiment. Data are representative of six independent experiments with similar results.

Cytokine measurements in the supernatant of activated CD4^+^ monocytes derived from mice spleen revealed that IL23R-CHR significantly inhibited the secretion of IL-17a and IL-22 (Figure 3). Additional, when IL23p40 mAb was used a positive control, the results indicated the effectiveness of the two proteins (Figure 3). IL-23 was reported to promote IL-17 secretion by promoting Th17 development [24]. However, in our experiments the IL23R-CHR appeared to down-regulate the IL-17a level *in vitro* under Th17 polarization condition in a dose-dependent manner. This indicated that the soluble IL23R-CHR protein could antagonize the binding function of endogenous IL23R and block the IL-23 activity, being consistent with the previous reports.

The activation and differentiation of native CD4^+^ lymphocytes into Th1 or Th2 cells require both TCR/MHC-peptide specific recognition and co-stimulatory signals. Antibodies specific for the TCR–CD3 complex (aCD3e) provide an initial activation signal, and aCD28 molecule usually serves as a co-stimulatory signal [25]. However, about the precise role of aCD28 as a co-stimulatory molecule remains controversial in the regulation of Th17 proliferation [26], [27].In our experiments, because aCD28 (1 ug/ml) inhibited the secretion of IL-17a (about 1.8 fold), our Th17 polarization condition included aCD3e for CD4+ T cells activation, hTGFβ, IL-6 for Th17 differentiation, and IL-23 for Th17 development, proliferation.

**Figure 4:**
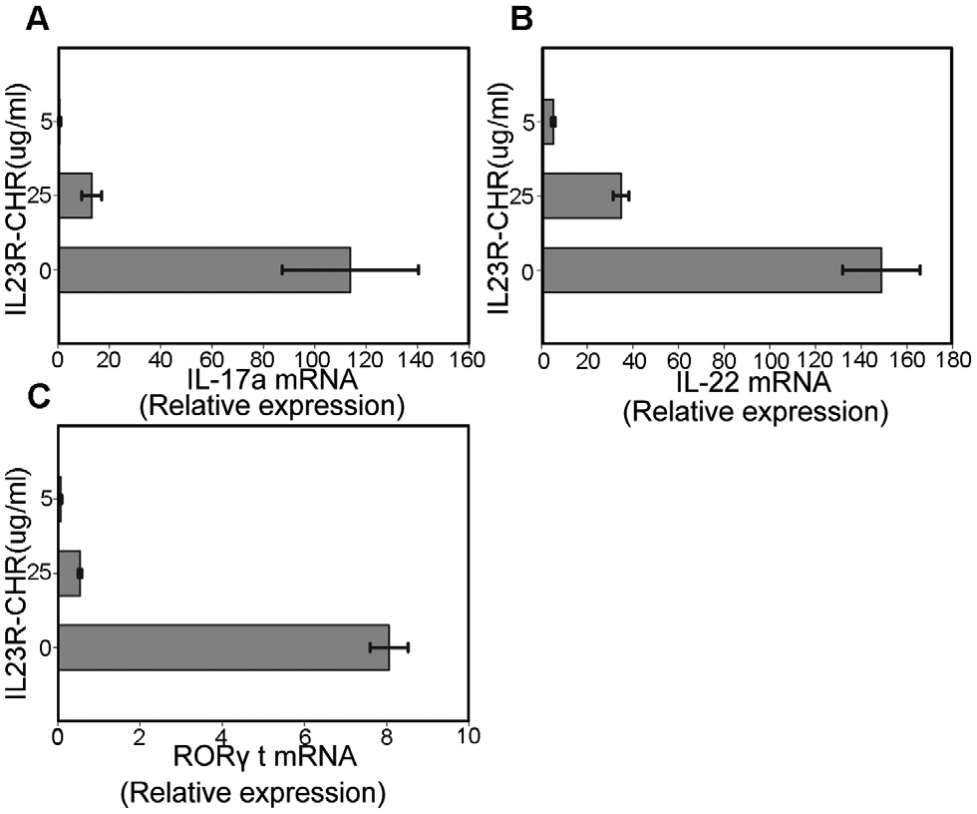
IL23R-CHR inhibits the expression of Th17 related mRNA. A, B and C show the results of Quantitative RT-PCR of the expression of mRNA encoding RORγt, IL17a and IL22 in Th17 cells treated with/without IL23R-CHR. Values are normalized to their average beta-actin values and are presented as relative expression units. Error bars indicate ± SD among duplicate samples from one experiment. Data are representative of three independent experiments with similar results.

Since RORγt expression directly induces IL-17a secretion, the effects of IL23R-CHR on RORγt were further evaluated by Q-PCR. It was observed that IL23R-CHR was able to suppress RORγt and IL-17a mRNA level in CD4^+^T cells (Figure 4). Meanwhile, the effects on another Th17 cytokine, IL-22, was also investigated, and similar results were obtained for IL-22 protein level in activated cell supernatant and IL-22 mRNA level in activated cells, in which they were both significantly decreased in the presence of IL23R-CHR.

### IL23R-CHR Treatment Significantly Suppressed Th17 Differentiation in vitro

**Figure 5:**
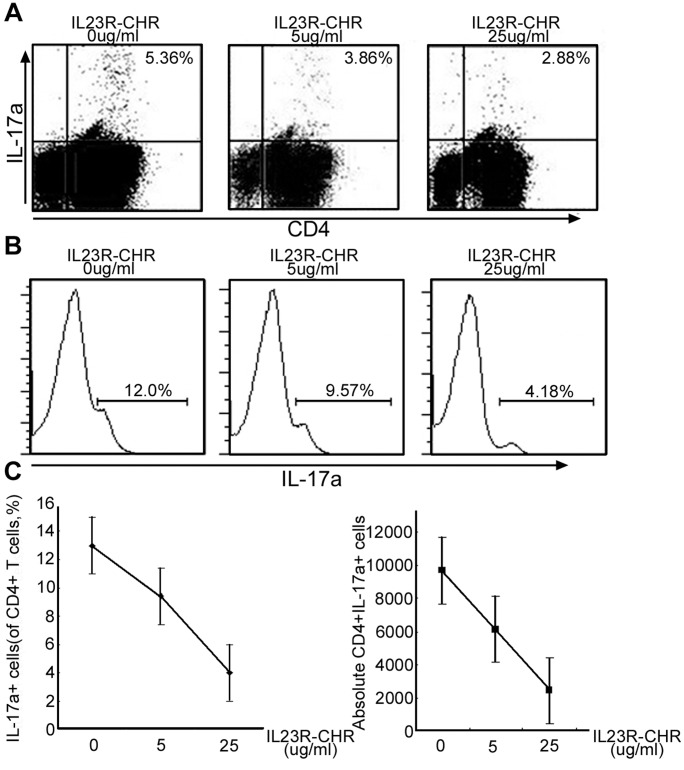
IL23R-CHR suppressed the differentiation of Th17 cells. A, B and C are Flow cytometry analyses of Th17 development in CD4+T cells cultured in the presence of IL23R-CHR. Naive CD4+ T cells from C57/BL6 mice were purified by CD4+ magnetic beads sorting, and stimulated with pre-coated anti-mCD3e (5 ug/ml) under Th17 polarizing condition (hTGFβ, IL-6 plus IL-23). Purified IL23R-CHR of different concentrations was added at the beginning of the cultivation. After 72 hours, cells were stained for surface CD4 antigen and intracellular IL-17. Plots in A are gated on whole cultured cells. Histograms in B are gated on CD4+ T cells. Results of A and B are analyzed in C. Data are representative of three independent experiments with similar results.

The direct effects of IL23R-CHR on Th17 cell differentiation were analyzed by treating activated CD4^+^ T cells with the Th17 polarizing cytokines IL-6, TGF-β and IL-23. The Th17 differentiation rate of purified CD4^+^T cells isolated from C57/B6 mice was markedly decreased compared to T cells treated with IL23R-CHR. (Figure 5A). By the increase of IL23R-CHR concentrations (0 ug/ml, 5 ug/ml and 25 ug/ml), the percentage of IL-17a^+^ cells in CD4^+^T cells decreased (12%, 9.57% and 4.18% respectively) (Figure 5B, C). The absolute number of Th17 cells (IL-17a^+^CD4^+^ T cells) in the activated sorting cells was also counted to decrease by around five fold (0 ug/ml, 10000 cells to 25 ug/ml, 2000 cells). IL-23 was reported to stabilize the Th17 phenotype [28], however, our study demonstrated that the blockage of IL-23 signals with IL23R-CHR protein resulted in significant suppression of Th17 differentiation.

## Discussion

Th17 cells belong to a novel T helper cells subset that mainly expresses IL-17 during inflammatory responses [29]. Th17 differentiation can be induced by IL-6 and TGF-β through RORγt, leading to the production of IL-17a and IL-22. Differentiated Th17 cells are further stabilized and amplified by the actions of IL-23 [30], [31]. Evidence presented in the present study suggested that IL23R-CHR functions as a rescuer during Th17 development, causing inhibition of RORγt expression and suppression of Th17 related cytokine expression.

The IL-23 receptor complex which composed of IL23R and IL12β1 is a type I transmembrane protein, human IL23R cDNA encodes a 629 amino acid (aa) with a 23 aa residue signal peptide, a 332 aa residue extracellular domain, a 21 aa residue transmembrane domain and a 253 aa residue cytoplasmic region. The human IL23R also contains an N-terminal Ig like domain, two FNIII domains in the extracellular domain. Human IL23R has a WQPWS sequence in the transmembrane proximal cytokine receptor domain similar to the cytokine receptor signature WSXWS motif, often called two cytokine receptor domains or cytokine binding homology region (CHR). Based on the structural characteristics, we amplified the gene of the “two cytokine receptor domains”, and named it as IL23R-CHR. Human and mouse IL23R share 66% amino acid sequence identity. They both contain a WQPWS sequence similar to the cytokine receptor signature WSXWS motif [16]. Mouse IL23R is expressed in mouse T helper cells, bone marrow, dendritic cells and macrophages. On the other hand, IL23R-CHRis expressed on the cell surface to recognize and respond to IL-23 by the WQPWS sequence. Up to now, both the extracellular and intracellular domains of IL-23R have been reported, but the CHR domain has hardly been analyzed, soluble receptors consisting only of the extracellular part are potent inhibitors of ligand activity. They bind the ligands with the same specificity and affinity as the membrane bound receptors without eliciting an intracellular signals. Our data showed that the exogenous soluble human IL23R-CHR protein could bind with human/mouse IL-23 complex and inhibit the binding of mouse IL-23 to endogenic mouse IL-23 receptor complex on CD4^+^T cell surface *in vitro*.

**Figure 6:**
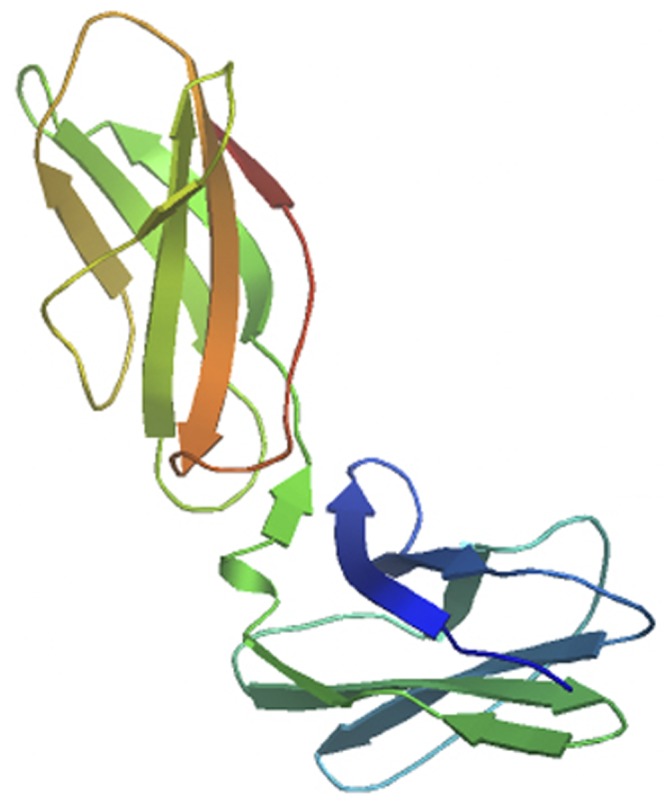
Cartoon structure of IL23R-CHR. The cartoon structure of IL23R-CHR was created by homology modeling based on template (PDB ID 1i1r) using Swiss-model online modeling service. The conformation of IL23R-CHR is predicted to be dominated by β sheet.

In this study, we created a 3D structure of IL23R-CHR by homology modeling based on template (PDB ID 1i1r) using Swiss-model online modeling service (Figure 6). The secondary structure was predicted to be dominated by β sheet, and our circular dichroism spectra confirmed the prediction. Although IL23R-CHR showed a good binding affinity to IL-23 complex *in vitro*, our results are not all the same with a previous report [16], which described that hIL23R-Ig could not bind to human IL-23 *in vitro*, and could not act as an effective antagonist of IL-23. However, in later publication [32], hIL23R-Ig was found to bind human IL-23 using competition ELISA and had a good affinity. Additionally, Yu et al [33] demonstrated that a naturally occurring IL23R variant Δ9 was able to bind human IL-23 *in vitro*. In our binding assays, native PAGE showed that IL23R-CHR bind to IL-23 in a dose-dependent manner, and direct binding ELISA assay measured the binding affinity to be around 90 nM. Furthermore, we observed that Th17 cells differentiation level was significantly down-regulated by targeting IL-23 with soluble IL23R-CHR protein, which was consistent with the results in IL23R deficient mice [18]. Moreover, we also demonstrated that IL23R-CHR mediated-Th17 suppression was through blockage of IL-23 signals on CD4+ T cells, resulting in lower RORγt expression, and therefore lowering the IL-17/IL-22 expression. The cytokines assays by intracellular staining and ELISA suggested that IL23R-CHR can inhibit naive CD4+ T cells polarizing into Th17 cells in a dose-responsive manner.

IL-23 regulates Th17 development and adjusts IL-23/IL-17 inflammation axis by controlling the expression of many Th17 related genes. Thus, to understand the molecular mechanism involved in differentiation of Th17 cells, we used quantitative PCR to evaluate mRNA levels of RORγt and other molecules implicated in Th17 differentiation. As a result, the repression of RORγt, IL-17a and IL-22 mRNA was observed. Yu et al [33] explained that IL-23 signals could be blocked to result in the inhibition of STAT3 phosphorylation, and the critical role for STAT3 expression in Th17 development has been described [34], [35], [36] since IL-23 induces a positive feed back loop in terms of IL23R expression and for further IL-23 responsiveness, STAT3 activation is required for IL23-mediated induction of its own receptor. Consequently, IL23-induced activation of STAT3 also plays an important role in IL-17 production. In this study, compared to the Th17 induced group, we postulated that pSTAT3/STAT3 in IL23R-CHR treatment group could be down-regulated, leading to the suppression of endogenic IL23R and therefore disturbance of the feed-back-loop of IL-23, which remains futher clarification.

**Figure 7:**
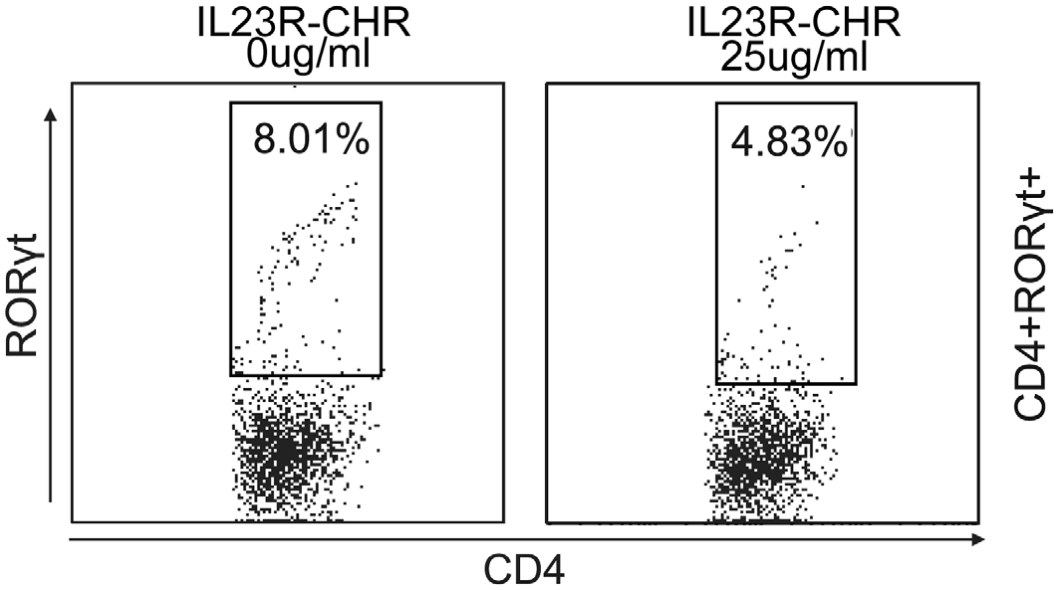
CD4+RORγt+ cells were repressed in the presence of IL23R-CHR. Naive CD4+ T cells from C57/BL6 mice were cultured under Th17 polarization condition with different IL23R-CHR concentrations. After 72 hours, cells were stained for surface CD4 antigen and intracellular RORγt, plots are gated on CD4+ T cells.

RORγt has been considered as a master-regulator to direct the differentiation of Th17 cells and IL-17a production. Deficiency in RORγt results in diminution of Th17 activity and severe reduction of IL-17a [37]. Yu et al [33] described the ability of a soluble endogenous external domain of the human IL-23R to inhibit human Th17 development *in vitro*, while we acknowledge the similarity between Yu’s work and our present findings, we do have a different result on RORγt. We found that the mRNA level of RoRγt in mouse Th17 cells were repressed under the treatment of IL23R-CHR *in vitro,* also our RORγt intracellular staining results demonstrated that CD4+RORγt+ cells were repressed in the presence of IL23R-CHR (Figure 7), but in their report, RORγt was not influenced in human Th17 cells *in vitro*. IL-23/RORγt interaction between human and mouse is most likely different, Cornelissen et al [38] found that IL-23 regulates RORγt in CD4^+^ T and TCRγσ^+^ T cells, RORγt was decreased in CD4^+^ T cells from IL-23p19KO mice compared to WT mice, indicating that the interaction between IL-23 and RORγt is related to species used in the experiments. Therefore, the IL23R-CHR mediated suppression of IL-17 production and Th17 cells differentiation *in vitro* could be the result of lower RORγt level from lower pSTAT3 expression.

The IL-23/IL-17 axis has been implicated in various autoimmune inflammatory disorders. IL-23 receptor blockage by neutralizing antibodies or soluble receptor proteins leads to decrease maturation and costimulatory molecules of DCs *in vitro* and *in vivo*. In addition, IL-23 promotes Th17 cells to produce high levels of IL-23/IL23R and promotes subsequent Th17 response. Blocking this IL23-dependent Th17 differentiation by IL23R-CHR should result in a reduction of Th17 cells, contributing to a protection against Th17 differentiation *in vitro*.

In summary, we have shown that IL23R-CHR, a truncated IL23R extracellular domain, was able to bind IL-23 *in vitro* and capable of blocking IL-23/IL-17 pathway and suppressing IL-23 mediated production of IL-17a through a STAT3-RORγt pathway *in vitro.* Our data suggested that IL23R-CHR could be an attractive strategy for designing novel therapeutics against IL23/IL17-mediated inflammation. In addition to the therapeutic potential of IL23p40 in autoimmune diseases, as demonstrated in our study, targeting a functional IL23R-CHR in IL23 receptor complex could also provide a useful route for ameliorating IL23/IL17-involved inflammatory diseases.

## Materials and Methods

### Construction, Expression, and Purification of IL23R-CHR

Human IL23R-CHR (amino acid residues 124–313) was cloned by Q-PCR from human spleen cDNA library (Biomics, China) using the following primers: 5′-GCCCATGGCTCCGCCAGATATTCCTGATG-3′ and 5′-CAGCTCGAGTTAATGAAAAAACGGTGAGCTCCA-3′. After purification with Tianquick midi purification kit (Tiangen, China), the IL23R-CHR gene was digested with *NcoI and XhoI*(takara) and inserted into pET32a following the manufacturer’s instructions (Novagen, Germany). The IL23R-CHR protein was successfully expressed in *E.coli* BL21 (DE3) (Novagen Germany) cells and purified by the following procedures. *E.coli* BL21 (DE3) cells were transformed with the pET32a/IL23R-CHR and the positive clones were selected by ampicillin resistance and confirmed by DNA sequencing. The selected positive transformants were grown in 500 ml Luria-Bertani medium containing 100 ug/ml ampicillin at 16°C for about 12 h until the absorbance at 600 nm reached 0.6.Subsequently, the transformants thus obtained were induced with 0.2 mM isopropyl-β, D-thiogalactoside (IPTG, Bio Basic, CA) for 4 h, harvested by centrifugation (10,000 g, 10 min, 4°C), and resuspended in 50 ml equilibration buffer (20 mM PBS,500 mM NaCl, 10 mM imidazole, PH7.4). After 10×10 s ultrasonic pulses, the suspension was centrifuged (10,000 g, 20 min, 4°C) and the clarified lysate was filtered through a 0.45 um filter. The crude protein mixture was then loaded onto a Ni-NTA column (5 ml packed, GE healthcare, USA) which was pre-equilibrated with equilibration buffer at a flow rate of 1 ml/min. The column was washed with 10 column volumes of wash buffer (20 mM PBS, 500 mM NaCl, 50 mM imidazole, PH7.4), then with 10 column volumes of cleavage buffer (50 mM Tris-HCl, 1 mM CaCl_2_, 0.1% Tween-20, PH8.0).The IL23R-CHR protein bound to the Ni-NTA column was digested by enterokinase (0.5 mg/1U, Sigma, USA) at 25°C for 16 h, and IL23R-CHR was released by wash with equilibration buffer. The eluted protein was collected and dialyzed overnight against PBS at 4°C.

### IL23R-CHR Binding Assays

Binding of IL23R-CHR protein to human IL-23 was evaluated by using non-denaturing polyacrylamide gradient gel under blue native PAGE conditions. Human IL-23 (R&D, USA) was diluted to 10 ug/ml, and 20 ng human IL-23 was incubated with IL23R-CHR with a mole ratio of 4∶1,2∶1,1∶1,0.5∶1,0.25∶1,0.125∶1 (IL23R-CHR:IL-23) respectively at 37°C for 2 h. The protein mixture was then separated by using non-denaturing polyacrylamide gradient gel which was then fixed and silver stained.

Binding affinity of IL23R-CHR protein to human IL-23 was examined by direct binding ELISA. 10 ng/well human IL-23 was coated in a 96-wells ELSIA plate (Costar, USA), and various concentrations of IL23R-CHR were added into the pre-coated plate, the IL-23-bound IL23R-CHR was detected by HRP labeled IL23R antibody for each IL23R-CHR concentration, IL23R mAb was pursued from R&D and labeled by HRP labeling kit (Thermo scientific, USA). IC_50_ values were determined by non-linear regression.

### Purification of CD4^+^ T cells

All experimental procedures with animals used in the present study were in accordance with the Guide for the Care and Use of Laboratory Animals as adopted and promulgated by the United States National Institutes of Health, and had been given prior approval by the Jiangsu Provincial Experimental Animal Manage Committee under Contract 2007(su)-0025.

Suspensions of single spleen cell were prepared from C57/BL6 mice (SPF, Comparative Medicine Centre of Yangzhou University, China).The spleen was teased through a sterilized 70 um nylon cell stainer (BD bioscience, USA) to obtain single cell suspensions in RPMI 1640 (contains 10% FBS, invitrogen, USA). Red blood cells were lysed with RBC lysis buffer (ebioscience, USA). CD4+ T cells were purified by using anti-CD4 magnetic beads (Miltenyi biotech, Germany).

### Th17 cells Polarization of CD4^+^ cells in vitro

Purified CD4^+^ T cells were prepared using magnetic beads for positive selection. The CD4^+^ T cells were plated at a concentration of 1×10^6^ cells/ml in 96-well plates pre-coated with anti-mCD3e mAb (5 ug/ml, 50 ul/well, ebioscience) and incubated for 3 days in the presence or absence of anti-mCD28 mAb (1 ug/ml, ebioscience), mIL-6 (10 ng/ml, R&D), hTGFβ (1 ng/ml, R&D), mIL-23 (10 ng/ml,R&D) (for Th17 differentiation). And the Th17 polarization cells were treated with or without IL23R-CHR (5 ug/ml, 25 ul/ml), and P40 mAb (1 ug/ml) was set as a positive control.

### Cytokine ELISA

At day 3, following activation, the cell supernatants were collected by centrifugation (10,000 g, 5 min), and were analyzed for secreted mIL-17a and mIL-22 by ELISA. All ELISA kits were pursued from Biolegend (USA), and the protocol per the manufacturer’s instructions.

### Real-time PCR Analysis

Total RNA was extracted from the cell pellet by using RNAprep pure cell kit (Tiangen) and cDNA was synthesized using Tianscript RT kit (Tiangen). The primer pairs used for Q-PCR assays were IL-17a forward, 5′-cagggagagcttcatctgtgt-3′, and IL-17a reverse, 5′-gctgagctttgagggatgat-3′; and IL-22 forward, 5′-tttcctgaccaaactcagca-3′, and IL-22 reverse, 5′-tctggatgttctggtcgtca-3′; and RORγt forward, 5′-acctcttttcacgggagga-3′, and RORγt reverse, 5′- tcccacatctcccacattg-3′. The quantitative RT-PCR was performed by using SYBgreen (Applied biosystem, USA) according to the manufacture’s instructions (Stepone plus, Applied biosystem).

### Intracellular Cytokine Staining for Flow Cytometry

Following the initial activation after 72 h, the cells were re-stimulated with GolgiStop (1 ul/ml, BD bioscience); PMA (20 ng/ml, sigma) and Ionomycin (1 ug/ml, sigma) for another 4–6 h. Surface and intracellular antigen staining were performed according to the manufacture’s instructions (BD cytofix/cytoperm, BD bioscience). All staining procedures were using directly conjugated mouse mAbs: PE-antiCD4, APC-antiCD4 (BD bioscience), Alex flour647-IL17a, APC-RORγt (ebioscience). Cellular staining was measured on a FACS Calibur instrument (BD bioscience) and data were analyzed using Flowjo software (Treestar int, USA).
